# Psychological intervention – a critical element of rehabilitation in chronic pulmonary diseases

**Published:** 2014-06-25

**Authors:** O Popa-Velea, VL Purcarea

**Affiliations:** * Department of Medical Psychology, "Carol Davila" University of Medicine and Pharmacy, Bucharest; ** Department of Healthcare Marketing, Technology and Medical Devices, Medical informatics and Biostatistics, "Carol Davila" University of Medicine and Pharmacy, Bucharest

**Keywords:** rehabilitation, psychological, psychotherapy, chronic pulmonary diseases

## Abstract

Abstract

Chronic pulmonary diseases represent a segment of pathology with an increasing prevalence worldwide, this requiring joint efforts from specialists in this field to (a) identify those factors insufficiently explored so far, but critical for their evolution and (b) address them via new therapies. This study aims to explore the existing data regarding the psychological factors involved in the dynamics of chronic pulmonary diseases and the main possibilities of psychological intervention, as a distinct part of pulmonary rehabilitation (PR). 49 articles published on this topic in peer-reviewed journals between 1979 and 2010, indexed in PubMed, ProQuest and EBSCO databases, were examined for evidence. Among psychological factors considered important by study authors were the following: 1) the deficient instruction of the patient, 2) decreased treatment motivation, 3) a marginal social role, 4) a disadaptive cognitive style and 5) psychiatric comorbidity (especially anxiety and depression). Efficient interventions were, for physicians, 1) patient education and 2) designing a personalized self-management plan, and for the clinical psychologists, 1) cognitive-behavioral therapy, 2) biofeedback, 3) family therapy, 4) relaxation and 5) hypnosis. Despite the undeniable effect of these methods in selected cases, the high heterogeneity of designs and personal affiliations of researchers do not allow new generalizations about their efficacy or their routine implementation into PR. Further research including larger samples, more uniform designs, construction of consensual international standards regarding the objectives of PR, and assessments done by experts from multiple study domains could contribute to a better understanding of the role psychological interventions could play in PR.

Abbreviations: COPD = chronic obstructive pulmonary disease; SES = socioeconomic status; PR = pulmonary rehabilitation; PEF = peak expiratory flow; CBT = cognitive-behavioral therapy; FEV1 = forced expiratory volume in one second

## Introduction

Chronic pulmonary diseases are among the most common somatic diseases, with chronic obstructive pulmonary disease and asthma holding majors position among all statistics of morbidity [**1,32**]. They also cause approximately 7% of all deaths worldwide and represent 4% of the global burden of the disease [**2**]. 

 The extension of chronic pulmonary diseases and their ever-increasing costs for society made the supplementation of classical pharmacological therapy with more modern rehabilitation programs necessary, which gained popularity in the last decades to the point where they have been incorporated in care, in the most developed countries. According to the American Thoracic Society and European Respiratory Society, pulmonary rehabilitation (PR) represents "an evidence-based, multidisciplinary, and comprehensive intervention for patients with chronic respiratory diseases who are symptomatic and often have decreased daily life activities" [**3**]. Its main aims include the decrease of symptoms, the optimizing of the functional status, the increase of participation, and the decrease of the health care costs, through stabilizing or reversing systemic manifestations of the disease. 

 Within the programs of PR, psychological and social support is generally considered an ingredient that is desirable, as it can "facilitate the adjustment process, by encouraging adaptive thoughts and behaviors, helping patients diminish negative emotions, and providing a socially supportive environment" [**3**]. However, the knowledge of the psychological factors involved in the development of chronic pulmonary conditions remains limited in many clinical settings and the use of psychotherapeutic intervention is still not systematic.

## Aim, method

This paper aims to make an overview of the main psychological factors involved in the dynamics of chronic pulmonary diseases and of the main possibilities of psychological intervention, as a distinct part of pulmonary rehabilitation. A number of 49 articles on these topics published in peer-reviewed journals between 1979 and 2010 and indexed in PubMed, ProQuest and EBSCO databases were examined to seek for evidence regarding these data. They are listed in the reference list with *.

## Results

Involvement of psychological variables and behaviors in the dynamics of chronic pulmonary diseases 

 The involvement of psychological variables and behaviors in the onset and evolution of chronic pulmonary diseases seems to be largely known, however, much less addressed by classical medicine. This is not only because of the traditional dualistic view of looking at the psyche and at the soma as two separate entities, but also because the impact of psychological factors on the evolution of the disease is often subtle enough to be overlooked for a long time, both by the patient and by the medical team. In fact, psychological variables should not be minimized anymore - and addressed as soon as possible - at patients in whom risky behaviors have become a matter of lifestyle, or compliance tends to be lower, on the expense of behavioral factors, irrespective of the quality of care. Clinicians should also take into consideration the fact that psychological dysfunctions are remarkably versatile and can intervene in the dynamics of the disease at a double level, either as an input (creating higher risks for getting or worsening the disease) or as an output (as a consequence of the long-term respiratory symptoms). Often, this creates the ideal conditions for a vicious circle, where somatic symptoms are at the same time the consequence and the premise of psychological deterioration. 

 Among psychological variables that can - and should be - the focus of physician’s attention, the most important are the following: 

 Educational factors (the deficient instruction of the patient) 

 Low instruction has been proven to be important especially in diseases with a chronic evolution and an important epidemiological impact, such as COPD, asthma and tuberculosis [**4-7**]. Despite educational programs centered on improving awareness of early disease symptoms and high-risk behaviors, the mere symptom recognition remains a challenge for some patients [**8**]. More personalized programs that should take into account (a) the cultural background of the patient, (b) the information that is assessed as significant for the afflicted person and (c) the desire to control the disease, seem more than necessary. These programs should consider emphasizing the role of personal responsibility, especially in low educated individuals, as they tend to leave all the responsibility for the disease in the doctor’s hands. 

 Decreased treatment motivation 

 Low motivation for treatment can stem not only from previous real or imaginary treatment failures, but also from the patient’s false representations on the cost-benefit ratio of consulting the doctor or initializing / continuing a therapy. Inner motivation is generally critical for a better disease outcome [**9**], as patients with inner motivation will be able to confront more successfully with the challenges and obstacles of the disease (such as a long-term or a costly treatment). This is why the physician (alone or as a part of a team containing a health psychologist) must identify those disease representations that impair a satisfactory inner motivation and address them. Techniques such as active listening, showing empathy and encouraging of positive transference are generally useful. Still, in some cases, motivational decrease remains a difficult target, especially in certain age groups (e.g. elderly; teens [**10**]) or because the decrease of motivation and the necessary action stemming from it are not followed by immediate negative consequences. 

 Taking on a marginal social role 

 The low socioeconomic status (SES) is frequently associated with non-compliance (through the restriction of access to quality health care or to primary health care [**11**], late presentation to the doctor, early resignation), but also with behaviors with direct pathogenic implication (e.g. smoking, alcoholism). Many of these patients often develop a vicious circle that can darken the prognosis: a disease with a social impact (e.g. tuberculosis) can lead to marginalization / stigma, decreased compliance, persistent risk behaviors, which in turn worsen the disease and can offer reasons for even higher punishment / marginalization / labeling. 

 Sometimes the marginal social role does not stem from a low SES, but from the patient’s insufficient ability / desire to find and use social support. Not only individuals with schizoid personality traits pertain to this category, but also normal persons, for example those with an avoidant or anxious attachment style. Quite often, they encounter difficulties in obtaining useful advice regarding the best ways to better manage their more critical symptoms. An unsupportive family can discourage the expression of symptoms, this leading to decreased compliance [**12**] and a worse prognosis. In addition, in couples with problems, stressful conversations may cause a poor management of symptoms, or their amplification, via increased anxiety. 

 Disadaptive cognitive style 

 Poor levels of self-efficacy, sense of coherence, hardiness and optimism are often connected to smaller trust in the medical intervention, but also to self-harmful behaviors, passivity, low health-related quality of life and a lower compliance [**13-16**]. This can be either a direct relationship, but also an indirect one, mediated by psychiatric comorbidity [**17**]. 

 Psychiatric comorbidity 

 The existence of psychiatric disorders, even mild, can significantly influence the attitude towards the pulmonary disease and, consequently, its prognosis. 

 Anxiety may play a direct pathogenic role in afflictions such as asthma and COPD, as it commonly triggers or aggravates tachypnea and dyspnea. Anxious patients are also prone to overmedication, by misinterpreting their symptoms as the result of an aggravation of their pulmonary disease [**18,19**]. This can also be misleading for untrained pulmonologists [**20**]. 

 Long-term consequences of persistent anxiety are important, as generally, this symptom is associated to greater disability, poorer functional status, and increased length of hospitalization [**21**]. 

 Depression is not a rare premise or outcome in chronic pulmonary diseases. For example, in COPD its prevalence ranges from 37% to 71% [**22**], which is 2.5 times higher than in the general population [**23**]. The risk of depression is directly proportional to the irreversibility of symptoms, their psychological impact and the associated decrease in the patient’s quality of life. Depression can be important also for the relatives of the patient, and is generally proportional to the degree of patient disability [**24,25**]. 

 Being depressed is a well-documented risk for non-compliance and increased prevalence of risk behaviors [**26,27**]. Even in cases of subclinical depression, the prognosis of pulmonary disease can be influenced via medication underdosing, very frequently encountered in these patients [**28**]. 

 Interventions: the psychological components of pulmonary rehabilitation 

 Pulmonary rehabilitation provides a successful "opportunity for a collaborative care model between mental health professionals and a multidisciplinary pulmonary team in chronic pulmonary diseases" [**21**]. This is also a rather inexpensive and well-tolerated alternative to the pure pharmacological treatment [**29,30**]. 

 Among the classical methods of PR (that typically include exercise training, nutritional counseling and energy-conserving breathing techniques), educational and psychotherapeutic strategies can address those cases in which psychological factors have a large contribution in pathology and in the disease evolution. Several of these strategies are accessible to the doctor, whereas others are only to the clinical psychologist. 

 Interventions available for the physician and the medical team 

 Patient’s education 

 The purpose of this component of PR is, in general, to provide the patient and his/her family with the information needed for the optimum management of the disease. However, educational programs aiming only patient knowledge proved not improve enough the health status [**31,32**]. More efficient educational programs should also include elements such as a better understanding of the therapy (thereby inducing the idea that it is a continuous process); distributing relevant information for the patient; discussing expectations and apprehensions of the patient; encouraging the patient to express his/her perception of the disease; developing a real partnership between the doctor and the patient; emphasizing treatment benefits; enhancing the trust in the physician; cultivating patient autonomy. 

 An important element of the educational strategy is its adjustment to the socio-psychological profile of the patient. A regular review of the therapeutic program with the patient and a frequent reinforcement of certain behaviors are also at the core of an efficient educational program. 

 In all chronic pulmonary diseases, these general goals should be blended with specific goals. For example, in the particular case of asthma, the essential data to be communicated to the patient include information about early identification of precipitating or triggering factors of the disease; about main differences (indications, possible adverse reactions) between control and crisis medication; information to facilitate the training of the patient regarding the correct use of medication (especially the inhaled type) and (if necessary) of the PEF-meter; data that allow the timely identification of signs of aggravation in asthma; the criteria, useful for the patient, for consulting a doctor in crisis situations; information which could motivate the patient in correctly using the preventive medication; and details that would respond to the patient’s misunderstandings, fears and possible skepticism [**33**]. 

 Personalized plan for the self-management of the disease 

 It aims the individualization of the treatment and of the lifestyle recommendations to patient’s essential psychological characteristics. This approach is considered consistent with Leventhal’s multifactorial cognitive model [**34**], which claims that the patient’s interpretation of the symptoms and of the treatment, the patient’s coping style and the adequacy of the treatment to patient’s core values are key factors in understanding the attitude versus the disease and the treatment. Within this paradigm, besides explaining the need for therapy, the clinician should give importance to elements such as the exploration of the way the patient weighs the benefits and the disadvantages of the treatment, or to how well the restrictions during the treatment are compensated by the benefits in the health status. 

 Kolbe [**35**] claims that the efficiency of a self-management personalized plan can be assessed by measuring five of its parameters (**Table 1**). 

**Table 1:** Essential elements of a personalized self-management plan (Kolbe, 2002; modified)

Element	Content
Relevance	The message addressed to the patient must be adapted to his/her cultural level, medical knowledge and attitudes, beliefs and interests. This can be done by keeping into the intervention plan those elements that are relevant and motivating at a given time, and by eliminating those that are or may become irrelevant, incomprehensible and disturbing for the patient;
Realism of goals	The objectives of the treatment plan (as they are formulated by the doctor) and the goals of the therapy (as perceived by the patient) should be similar and satisfactory for both partners of the therapeutic relationship. Treatment goals should be quantified in easily measurable and relevant parameters for both the doctor and the patient.
Availability	It is necessary to consider all the patient’s potential resources (including material, instrumental and emotional support)
Reinforcement	A long-term relationship between the physician and the patient is preferable, to ensure an effective reinforcement of good behaviors, maintenance of trust and compliance. Reinforcement technique should be tailored to the patient’s preferred relational style (more authoritative or more symmetrical).
Flexibility	Represents an essential element in the progress of such strategies and is probably directly connected to the idea of an "individual therapeutic plan". This means essentially to react promptly to incidents or accidents that happen on the way, as well as to the favorable developments, and to work with a certain array of scenarios that allow the therapist to assess, at any time, the way the disease is experienced by the patient

 Disease management programs conducted on these premises are generally found to be remarkably effective. According to Clark et al. [**36**], they lead to a lower symptom score and a significantly reduced number of emergency calls.

 For the particular case of asthma, previous research done in the Romanian care system suggests that a well-designed personalized self-management plan can significantly improve care satisfaction and adherence [**37,38**]. At least three factors seem to be critical designing such a plan:

 - adequate and accurate information provided to the patient;

 - a type of doctor-patient relationship as close as possible to the one expected / desired by the patient;

 - ensuring a productive integration of the clinical psychologist in the care team and early addressing all patient’s psychiatric comorbidities.

 Interventions available to the clinical psychologists

 These are typically necessary in patients who have psychiatric comorbidity, but they can also address dysfunctional coping strategies, or the low sense of personal control on those symptoms that are mostly perceived as unconscious physiological processes, such as breathing movements. A clinical psychologist can be also helpful in setting up an individualized plan of disease management, given the greater familiarity of the psychologist with the risk factors related to one’s family, group or cultural affiliation.

 Cognitive-behavioral therapy (CBT)

It is a form of psychotherapy that aims, in its early phases, to replace the patient’s irrational beliefs (e.g. "there is no hope for me anymore") with more realistic thoughts, which would facilitate a better adaptation to the situation created by the disease. Later, CBT focuses on the disadaptive behaviors stemmed from the original dysfunctional thoughts and proposes new alternative ways of behavior. 

 The main strength of CBT lies in its potential to discontinue the multiple vicious circles created between the psychological and somatic symptoms, acting on at least three levels:

 - misevaluation of symptoms (in this sense, it addresses exaggerations, catastrophic perceptions and biased selection of illness clues); 

 - misinterpretations about the efficacy of treatment or preventive behaviors (it may modulate exaggerated expectations from these and facilitate the design of more realistic therapy objectives);

 - transforming of randomly efficient coping strategies into permanent ones.

 As a component of rehabilitation in chronic pulmonary diseases, CBT can be an effective option to address especially anxiety (panic attacks, panic-spectrum disorders) and depression, be them primary or secondary to the disease’s relapses [**39,40**]. A recent study also showed that CBT group treatment and COPD education have similar benefits on health-related quality of life [**41**]. Although more ample studies are still necessary to document the specific beneficial effect of CBT [**42**], two indirect advantages are already clearly outlined: it undeniably reduces the stress perceived by the patient during therapy and it makes the clinician pay more attention to the patient’s representations and concerns about the disease [**8**]. In turn, these effects can ameliorate prognosis and contribute to a higher patient compliance.

 Biofeedback

 This technique uses various visual and auditory instruments to teach the patient to manage different body functions, otherwise not accessible to voluntary control. In asthma and COPD, the biofeedback’s target is to achieve relaxation of specific muscles that have an additional role in symptoms of breathlessness and chest contraction [**43,44**]. So far, benefits in COPD seem more certain than in asthma [**45,46**], however more research is needed to evaluate the precise role biofeedback could play in PR. 

 Family therapy

 It addresses those familial factors that could trigger or maintain respiratory symptoms (e.g. asthma attacks) and be responsible for low compliance or risk behaviors.

 In asthma, six hours of family therapy per week have significantly improved, in 4 months of treatment, both the lung capacity of children suffering from asthma attacks, as well as the reported score of wheezing in these patients, in-between attacks [**47**]. Similarly, Weingartner et al. [**48**] found a significant increase of PEF in asthmatic children benefiting from family therapy. Gustaffson et al. [**49**] reported a significant increase of compliance and health-related quality of life in children attending family therapy.

 In COPD, family therapy seems useful especially for patients with long-term oxygen therapy, as this procedure challenges even more family normal roles and functioning. Authors found that poor self-identity, isolation from others and lack of flexibility in families with severe COPD can weaken the ability of the families to manage on a longer term in everyday life [**50**], thereby suggesting family therapy as a necessary approach.

 Relaxation therapy

 Typically, this comprises a series of psycho-physiological and imagination exercises, organized in three phases, which focus progressively on attaining a simple well-balanced breathing to the mastering of a psychological sense of well-being.

 On the somatic level, relaxation effects are remarkable in controlling symptoms such as dyspnea and tachypnea. In asthma and COPD, FEV1 improvement is constant and varies on average between 15% and 25% [**51,52**].

 Beyond the effect on the physiological level, relaxation can clearly bring an improvement of the psychological status, among which reduced anxiety, reduction or elimination of inappropriate emotional reactions, stimulation of confidence, self-mastery, and self-discipline [**53,55**].

 A clear advantage of including relaxation in PR is the fact that it is a technique, which is easy to access, implement and accept, this making its area of applicability quite wide.

 Hypnosis

 Hypnosis aims at modifying the patient’s normal state of consciousness and at introducing him/her – for a limited period of time – in an altered state, called "trance", characterized by a combination of relaxation and high suggestibility. In this state, the therapist can administer therapeutic suggestions, targeting both physical and psychological symptoms and shortcutting the normal defenses a chronic patient may develop during a long history of repeated failures in confronting the disease. Although the procedure is dependent on the suggestibility of the patient, its addressability remains large. For example, hypnosis can be implemented irrespective of the educational level of the patient, this being a substantial advantage for the management of those cases that could be exposed to non-compliance because of this particular reason.

 In pulmonary diseases, two important benefits of hypnosis are the decrease of anxiety (which in turn, diminishes the incidence of complications) and the increase of compliance (via increasing self-efficacy and decreasing catastrophic interpretations of the treatment’s side effects). Hypnosis may also lead to the abandonment of risk factors (e.g. smoking) [**56**]. 

 Despite these positive effects, hypnosis has been used less often as an adjuvant therapy in PR, in part because of the high specificity of this technique, which makes it improbable to be integrated in standard care. When combined with CBT, hypnosis can be very effective, as each of the two therapies target a different compartment of the psyche, CBT aiming at the rational and behavioral (conscious) side, while hypnosis at the emotional (largely unconscious) one.

## Discussion

This overview of selected literature provided reliable data to argue in favor of psychotherapy, personalized self-management plans and education as necessary ingredients of PR, with benefits both on somatic and psychological symptoms.

Still, it is plausible that the use of these methods will further remain a matter of scientific debate, especially because the number of studies dedicated to these topics is still scarce, and some of them provide contradictory or not enough evidence-based results. In this sense, the methodology and the personal affiliation of the researcher seem to play a key role, especially in reporting benefits or flaws of those methods that require a high level of specialization and can be implemented on a fewer number of patients (such as hypnotherapy). Other potential obstacles in assessing the benefits of psychological interventions in PR come from the lack of specialists that could integrate these techniques in various medical settings and socio-cultural environments. For example, education can be highly country-specific and the instruments of measuring its efficiency can be quite dependent on the objective of health politics in a given area and on the available financial resources. Insufficient integration of the health psychologist in the care team, especially since the beginning of therapy, also represents a potential problem in evaluating the effectiveness of psychological intervention in PR.

Despite these remaining issues, existing data suggest that, at least for a series of chronic pulmonary patients, psychological interventions are critical elements that significantly influence their prognosis and quality of life. 
